# Prospective association between handgrip strength in childhood and the metabolic syndrome score and insulin resistance indices in adolescence: an analysis based on the Ewha Birth and Growth Study

**DOI:** 10.4178/epih.e2025001

**Published:** 2025-01-02

**Authors:** Seunghee Jun, Hyunjin Park, Hyelim Lee, Hye Ah Lee, Young Sun Hong, Hyesook Park

**Affiliations:** 1 Department of Preventive Medicine, Ewha Womans University College of Medicine, Seoul, Korea; 2 Graduate Program in System Health Science and Engineering, Ewha Womans University, Seoul, Korea; 3 Clinical Trial Center, Ewha Womans University Mokdong Hospital, Seoul, Korea; 4 Department of Internal Medicine, Ewha Womans University College of Medicine, Seoul, Korea

**Keywords:** Hand strength, Metabolic syndrome, Insulin resistance, Cohort studies

## Abstract

**OBJECTIVES:**

Low handgrip strength (HGS) in children and adolescents might be associated with the risk of metabolic syndrome (MetS) and insulin resistance. This study prospectively evaluated the association between HGS in childhood and MetS in adolescence.

**METHODS:**

Based on data from the Ewha Birth and Growth Study, this study analyzed HGS at ages 7 to 9 and metabolic indices at ages 13 to 15. In total, 219 participants were analyzed. The risk of MetS was evaluated using the continuous metabolic syndrome score (cMetS), and insulin resistance was assessed using fasting blood insulin and homeostasis model assessment of insulin resistance (HOMA-IR). Relative HGS in childhood was determined by dividing HGS by body weight and categorized as sex-specific quartiles.

**RESULTS:**

This study found an inverse association between relative HGS levels in childhood and MetS and insulin resistance in adolescence. For each 1-group increase in relative HGS quartiles, cMetS (standarard [Std] β=-0.64, p<0.01), HOMA-IR (Std β=-0.21, p<0.01), and fasting blood insulin (Std β=-0.21, p<0.01) all decreased on average. These associations remained significant even after adjusting for confounding factors.

**CONCLUSIONS:**

Our study showed a prospective association between HGS in childhood and the risk of MetS and insulin resistance in adolescence. It provides significant epidemiological evidence, emphasizing the importance of efforts to increase muscle strength from a young age to mitigate the risk of MetS and insulin resistance in adolescence.

## GRAPHICAL ABSTRACT

[Fig f5-epih-47-e2025001]

## Key Message

This study found that higher childhood relative handgrip strength was inversely associated with metabolic syndrome and insulin resistance in adolescence. The findings emphasize the importance of enhancing muscle strength early in life to mitigate metabolic risks.

## INTRODUCTION

Metabolic syndrome (MetS) is defined by a cluster of conditions including abdominal obesity, high blood pressure, dyslipidemia, and elevated fasting blood sugar levels [1,2]. Insulin resistance is not only a characteristic of MetS but also a risk factor for the syndrome. The syndrome can elevate the risk of cardiovascular diseases due to the interplay of multiple risk factors [3-5]. MetS in adolescents may persist in adulthood. Preventing MetS is crucial, as it significantly increases the likelihood of developing serious health issues such as cardiovascular disease and type 2 diabetes [6,7].

According to a study examining the prevalence and risk factors of MetS in Korean adolescents from 2007 to 2018, the prevalence of MetS, as defined by the National Cholesterol Education Program Expert Panel and Adult Treatment Panel III standards, increased from 4.5% to 6.2%. Additionally, the incidence of hyperglycemia rose from 5.3% to 12.0% [8].

Exercise and physical activity are beneficial for maintaining health and preventing MetS [1,9]. Muscle strength can be assessed through various measures, including maximum weight per repetition, high jump, sit-up, and squat. Handgrip strength, in particular, offers a relatively straightforward method for determining muscle strength and is commonly employed in epidemiological studies [10,11].

Previous studies on adults have reported that low handgrip strength was associated with the risk of cardiovascular disease and diabetes [12,13]. Furthermore, low handgrip strength in adults is related to MetS. A meta-analysis showed an inverse association between handgrip strength and MetS (pooled odds ratio [OR] for handgrip strength [low vs. high], 2.59; 95% confidence interval [CI], 1.08 to 5.63), and also found a significant dose-response relationship (OR, 0.68 per 0.1 relative handgrip strength; 95% CI, 0.52 to 0.75) [14]. In addition, fasting insulin (β= -0.003, p= 0.017) and homeostasis model assessment of insulin resistance (HOMAIR; β= 0.003, p= 0.025) values showed an inverse linear relationship with relative handgrip strength [15].

Numerous studies have explored the link between handgrip strength and cardiovascular disease in adults. However, research focusing on this association in children and adolescents is limited, with most studies being cross-sectional [12-17] and few being longitudinal. Additionally, there is a lack of research on MetS and insulin resistance, both of which are intermediate risk factors for cardiovascular disease. Therefore, this study aimed to assess the prospective relationship between relative handgrip strength in childhood and the risk of developing MetS and insulin resistance in adolescence.

## MATERIALS AND METHODS

### Study participants

This study utilized data from the Ewha Birth and Growth Study, a cohort established with pregnant women who visited the Obstetrics and Gynecology Department of Ewha Womans University Mokdong Hospital between September 2001 and June 2006. The cohort specifically included women who sought prenatal care during the 24th weeks to 28th weeks of gestation. It comprised 940 children born to mothers who agreed to participate in the study, with longitudinal observations beginning at the age of 3 in 2005. Regular follow-up examinations were conducted at ages 5 and 7, and then at each subsequent age interval. These follow-up assessments included physical measurements, questionnaires, dietary surveys, and the collection of blood and urine samples. Detailed information about the cohort study can be found in relevant publications [18].

**Figure 1.**
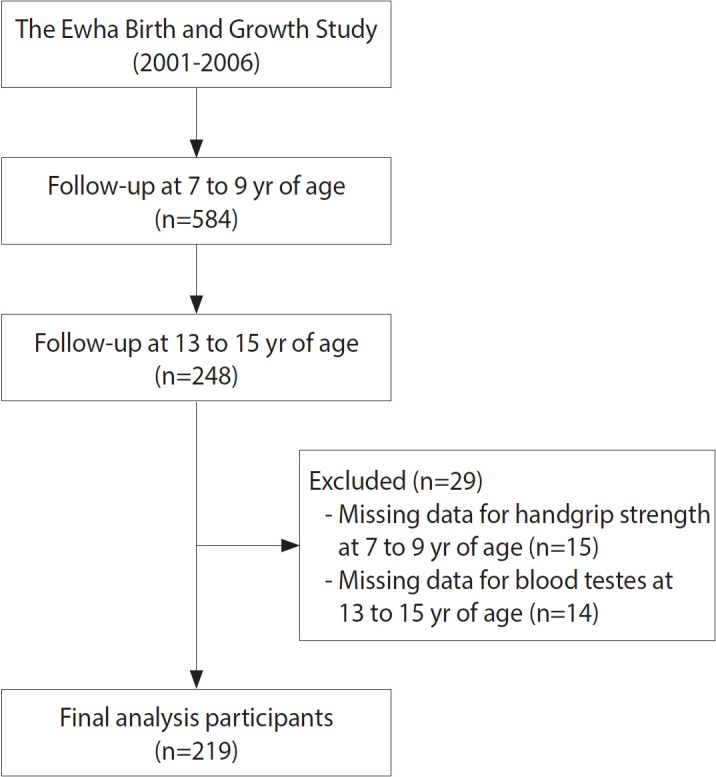
Flow chart of the study participants.

In this study, individuals were defined as study participants if they participated in at least 1 observation during the tracking period at ages 7-9 and attended all observations during the adolescent tracking period at ages 13-15. Of the 584 individuals observed at ages 7-9, 248 were considered study participants at ages 13-15. After excluding individuals with missing data on handgrip strength, MetS scores, and insulin resistance indicators, the final number of study participants was 219 (Figure 1).

### Measurement of handgrip strength

Handgrip strength was assessed during a tracking survey when participants were aged 7-9 years. Each participant stood, extended their arm straight and slightly to the side, and then forcefully pulled on a handgrip strength measurement device for 3 seconds using their dominant hand. Measurements were taken 3 times at intervals of 0.1 kg using the Lavisen Digital Hand Grip Dynamometer KS-301 (Lavisen Co., Ltd., Namyangju, Korea). The highest value recorded from the 3 attempts was used for analysis.

Since handgrip strength is correlated with physique, most studies evaluate relative handgrip strength to minimize its influence. This is calculated by dividing handgrip strength (kg) by body weight (kg) [17,19]. Therefore, we calculated relative handgrip strength and assessed it based on quartiles for both boys and girls [13,20]. For boys, the quartiles were defined as follows: first quartile (Q1< 0.35), second quartile (0.35≤ Q2< 0.40), third quartile (0.40≤ Q3< 0.48), and fourth quartile (Q4≥ 0.48). For girls, the quartiles were defined as first quartile (Q1< 0.33), second quartile (0.33 ≤ Q2 < 0.38), third quartile (0.38 ≤ Q3 < 0.43), and fourth quartile (Q4≥ 0.43).

### Measurement of metabolic syndrome score and insulin resistance indices

To assess the risk of MetS in adolescents aged 13-15 years, a continuous metabolic syndrome score (cMetS) was calculated [21,22]. This score included components such as body mass index (BMI), mean arterial pressure, triglycerides (TG), fasting blood glucose, and high-density lipoprotein cholesterol (HDL-C). Each component was standardized using the Z-score method, which accounts for mean, standard deviation (SD), and sex differences. The cMetS was derived by summing these components. Notably, the HDL-C component was multiplied by -1 before addition due to its inverse relationship with MetS risk [22]. A higher cMetS indicates a higher risk of developing MetS.

To assess insulin resistance, fasting blood insulin (FBI) and HOMA-IR values were examined. HOMA-IR is extensively used in clinical and epidemiological research due to its relatively high accuracy in evaluating insulin resistance [23,24]. Higher HOMA-IR values suggest a greater risk of insulin resistance [22].

### Covariates

Data on the general characteristics of the study participants were collected through questionnaires during the follow-up examination. Using data collected at ages 13-15, variables such as sex, age, average monthly household income, mother’s education level, and frequency of moderate physical activity were considered as confounding factors. These variables were selected based on a literature review [13,15,16]. The frequency of moderate physical activity was categorized into 4 groups: never, 1-2, 3-4, and ≥ 5 times/wk. Additionally, the change in BMI from ages 7-9 to 13-15 was used as a representative indicator of the participant’s growth.

### Statistical analysis

The normality of the distribution of participant characteristics was assessed. If the values conformed to a normal distribution, the mean and SD were reported; if not, the median and interquartile range were used. Due to the non-normal distribution of TG and FBI levels, a log-transformed analysis was utilized, with results presented after inverse transformation. Additionally, participants’ height, weight, and BMI were converted to standard deviation scores (SDS) using the 2017 Korean National Growth Chart for children and adolescents [25]. We employed general linear models to assess the impact of childhood relative handgrip strength levels on MetS score, its components, and insulin resistance in adolescence. Results were presented with adjusted means and 95% CIs, and multiple comparison analysis was conducted using the Tukey method. Linear regression analysis was also used to explore the impact of childhood relative handgrip strength levels on MetS components and insulin resistance in adolescence through trend analysis. These associations were further examined by sex, with all outcome variables standardized and considered. To improve the accuracy of the association between relative handgrip strength and metabolic outcomes, a sensitivity analysis was conducted using body fat mass (BFM) instead of body weight for calculating relative handgrip strength. Body composition, including BFM, was measured using an 8-electrode bioelectrical impedance analysis device, the InBody 230 (Biospace Co. Ltd., Seoul, Korea). All statistical analyses were performed using SAS version 9.4 (SAS Institute Inc., Cary, NC, USA), with statistical significance determined at a 0.05 level in 2-sided tests.

### Ethics statement

During follow-up, all participants and their guardians received a thorough explanation of the study, and written consent was obtained. The study received approval from the Institutional Review Board of Ewha Womans University Seoul Hospital (IRB No. SEUMC 2020-08-024).

## RESULTS

The participants in the study had an average height of 126.10 cm, weight of 26.42 kg, and a BMI of 16.44 kg/m^2^. The mean handgrip strength recorded was 10.47 kg, which corresponds to a relative handgrip strength of 0.40 when normalized to body weight. The growth status of both the included and excluded participants was comparable and consistent, as assessed using SDS. Furthermore, no significant differences in handgrip strength were observed (Supplementary Material 1).

A total of 219 participants—107 boys (48.9%) and 112 girls (51.1%)—were included in the study. At 7-9 years of age, boys and girls showed no significant differences in weight, BMI, or blood pressure, but TG and the insulin resistance indices were significantly higher in girls. At 13-15 years of age, boys were significantly heavier and the systolic blood pressure (SBP) was also higher, while girls continued to show higher TG levels (p= 0.04). In addition, the weight SDS was significantly higher in boys than in girls (p< 0.01), but the BMI SDS was similar between the sexes (p = 0.56). However, metabolic indicators, including the cMetS and insulin resistance indices, did not differ significantly between the sexes across both time points (Table 1).

Table 2 shows the relationship between the quartiles of relative handgrip strength at ages 7-9 and the MetS scores, MetS components, and insulin resistance indicators at ages 13-15. Significant differences in cMetS and BMI were observed based on the quartiles of relative handgrip strength (p= 0.01 for cMetS and p< 0.01 for BMI). The insulin resistance indicators, HOMA-IR and FBI, also showed significant differences according to relative handgrip strength quartiles (p< 0.01 for both HOMA-IR and FBI). In the results classified by sex, boys showed differences in BMI and log FBI, while girls showed differences in cMetS, BMI, and log-TG according to the quartiles of relative handgrip strength.

**Figure 2.**
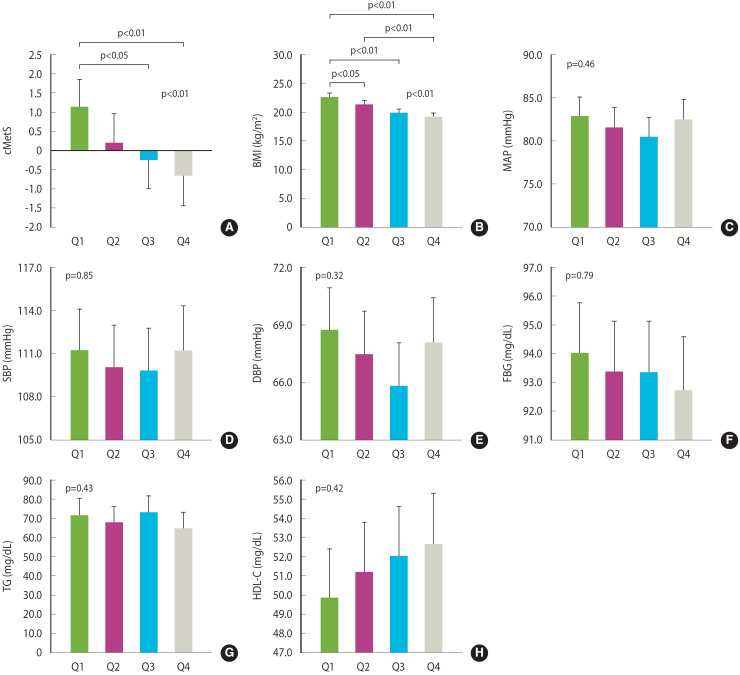
Effects of relative handgrip strength quartiles at 7-9 years of age on metabolic syndrome components at 13-15 years of age. (A) continuous metabolic syndrome score (cMets), (B) body mass index (BMI), (C) mean arterial pressure (MAP), (D) systolic blood pressure (SBP), (E) diastolic blood pressure (DBP), (F) fasting blood glucose (FBG), (G) triglyceride (TG), (H) high-density lipoprotein cholesterol (HDL-C). Results are presented as adjusted means with 95% confidence intervals (CIs). Adjusted mean with 95% CI was estimated under controlling for sex, age, monthly household income, mother’s education, moderate physical activity at 13-15 years of age, and change in BMI from the age of 7-9 to 13-15. Multiple comparisons were performed using the Tukey test.

**Figure 3.**
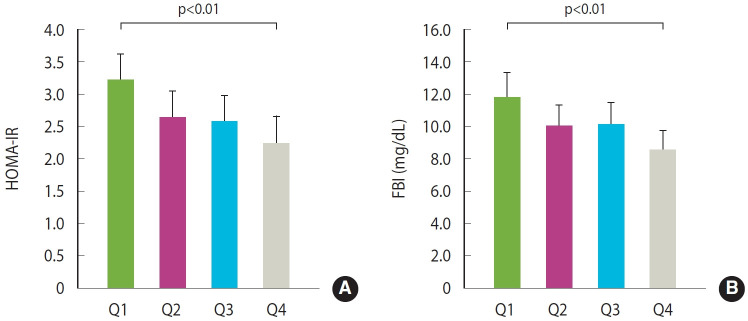
Effects of relative handgrip strength quartiles at 7-9 years of age on insulin resistance indices at 13-15 years of age. (A) homeostasis model assessment of insulin resistance (HOMA-IR) and (B) fasting blood insulin (FBI). Adjusted mean with 95% confidence interval (CI) was estimated under controlling for sex, age, monthly household income, mother’s education, moderate physical activity at 13-15 years of age, and change in body mass index from the age of 7-9 to 13-15. Multiple comparisons were performed using the Tukey test. Results are presented as adjusted means with 95% CIs.

We also assessed associations after adjusting for covariates. We presented the adjusted means for the MetS scores, its components, and insulin resistance indices based on the quartiles of relative handgrip strength (Figures 2 and 3). There was a significant difference in cMetS and BMI. Differences between the lowest and highest quartiles were evident (both p< 0.01), based on the Tukey post-hoc test. However, no statistically significant differences were found in other metabolic components. The adjusted mean difference between the lowest and highest quartile was also clear for HOMA-IR and FBI (p= 0.01 for HOMA-IR and p< 0.01 for FBI).

**Figure 4.**
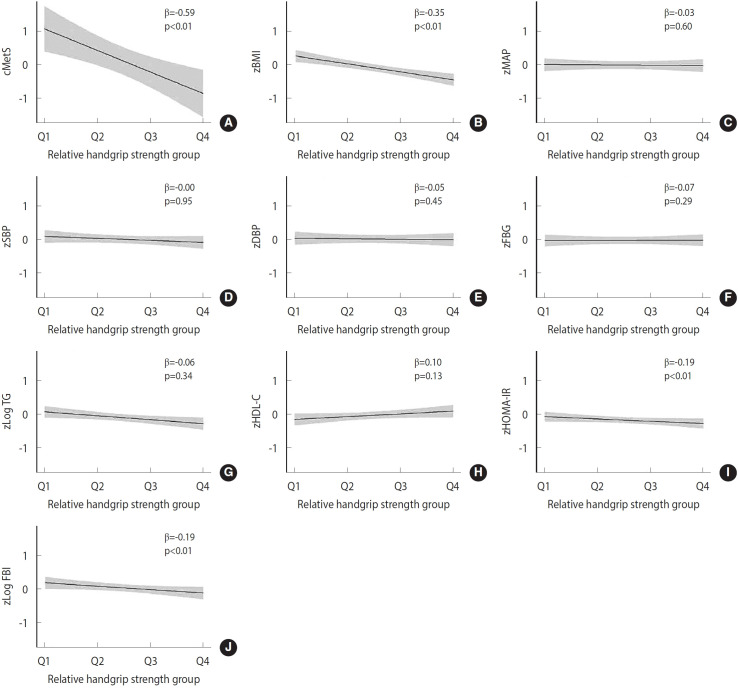
Linear regression results of childhood relative handgrip strength quartiles on metabolic syndrome components and insulin resistance in adolescence. (A) continuous metabolic syndrome score (cMetS), (B) body mass index (BMI), (C) mean arterial pressure (MAP), (D) systolic blood pressure (SBP), (E) diastolic blood pressure (DBP), (F) fasting blood glucose (FBG), (G) triglyceride (TG), (H) high-density lipoprotein cholesterol (HDL-C), (I) homeostasis model assessment of insulin resistance (HOMA-IR), and (J) fasting blood insulin (FBI). Relative handgrip strength quartiles were defined taking sex into account. The range of each quartile is as follows; 1st quartile (Q1<0.35), 2nd quartile (0.35≤Q2<0.40), 3rd quartile (0.40≤Q3<0.48), and 4 quartile (Q4≥0.48) in boys and 1 quartile (Q1<0.33), 2nd quartile (0.33≤Q2<0.38), 3rd quartile (0.38≤Q3<0.43), and 4 quartile (Q4≥0.43) in girls. Beta coefficients were obtained from linear regression model by assigning the median value to each quartile and treating it as a continuous variable. The model was adjusted for sex, age, monthly household income, the mother’s education level, moderate physical activity at the age of 13-15 years, and change in BMI from the age of 7-9 to 13-15.

We assessed the dose-response relationship between relative handgrip strength quartiles and the cMetS, components of MetS, and insulin resistance through linear regression analysis (Figure 4, Supplementary Material 2). As the relative handgrip strength quartile increased, the cMetS value significantly decreased by -0.64 (p< 0.01). After adjusting for confounding variables, the negative association between cMetS and relative handgrip strength quartiles remained significant (standarard [Std] β = -0.59, p < 0.01). Additionally, a similar trend was observed for the insulin resistance indices. Both HOMA-IR (Std β= -0.21, p< 0.01) and FBI (Std β= -0.21, p< 0.01) showed significant decreases with increasing relative handgrip strength quartiles. These associations remained significant after adjusting for confounding variables (HOMA-IR: Std β= -0.19, p< 0.01; FBI: Std β= -0.19, p< 0.01). Stratified analyses by sex showed results consistent with the main findings.

The sensitivity analysis results using BFM data showed that, in addition to the significant association shown in the original findings, HDL-C showed a significant association with relative handgrip strength (Supplementary Material 3).

## DISCUSSION

The findings of this study indicate an inverse association between relative handgrip strength in childhood and both MetS and insulin resistance in adolescence. Specifically, the associations between cMetS, BMI, HOMA-IR, and FBI persisted even after adjusting for various confounding factors.

Previous studies have lacked prospective assessments of the association between childhood relative handgrip strength and MetS in adolescence. However, the UP&DOWN study conducted in Spain, which included children aged 6-10 years and adolescents aged 12-16 years, demonstrated a consistent inverse relationship between handgrip strength levels and cardiovascular risk factors such as skinfold thickness, SBP, and insulin resistance at both initial and follow-up assessments [26]. Furthermore, research has investigated the connection between childhood physical fitness, including handgrip strength, and cardiovascular risk factors. A longitudinal European study targeting children aged 6-11 years, with a follow-up period of 2 years, indicated that lower fitness levels were linked to increased metabolic risk. In this study, handgrip strength was associated with metabolic risk, though significance was only observed in cross-sectional data [27]. Another study assessing the relationship between childhood muscle strength and the risk of MetS in adulthood found that children with higher muscle strength had a lower risk of developing MetS later in life, with reduced incidence rates observed during adulthood (risk ratio, 0.21; 95% CI, 0.09 to 0.49) [28]. These findings collectively suggest a potential link between childhood muscle strength, particularly relative handgrip strength, and the risk of MetS in both adolescence and adulthood. The results of this study corroborate those of previous research.

Insulin resistance is characterized by a reduced response of the body to the metabolic actions of insulin, which is essential for carbohydrate and lipid metabolism [29]. Therefore, insulin resistance is frequently considered a significant risk factor and a component of MetS [30,31]. Several studies have explored the relationship between handgrip strength and insulin resistance through prospective investigations. One study examining the link between childhood handgrip strength and adult insulin resistance revealed that a 3.57 kg increase in right handgrip strength among 9-year-old boys correlated with a 0.52 mU/L reduction in FBI and a 0.08 decrease in updated HOMA-IR (i.e., HOMA2-IR) in their adult years [32]. Furthermore, a cohort study that followed Danish adolescents for 12 years did not measure handgrip strength but discovered that adolescent muscle strength was inversely related to FBI and markers of insulin resistance in adulthood [33]. Our findings align with these previous studies.

Recent years have seen an increasing trend in the prevalence of MetS among both adults and adolescents in Korea, with rates reaching 22.9% and 4.8%, respectively [34,35]. Data from the Korea National Health and Nutrition Examination Study indicate that the prevalence of insulin resistance among Korean adolescents, assessed using HOMA-IR, was 9.8% [24]. Additionally, the National Health Insurance Service reported that the prevalence of type 2 diabetes among adolescents aged 10-14 and 15-19 in 2016 was 2.8 and 9.9 cases per 10,000 individuals, respectively, marking a significant increase from 2002 [36]. Moreover, the prevalence of hyperglycemia was reported at 11.7 cases per 100 individuals [37]. These trends highlight a growing risk of metabolic disorders, which could potentially lead to adult-onset diseases, underscoring the importance of prevention and intervention efforts. However, the 2022 Korean Youth Risk Behavior Survey revealed that the rate of physical activity among Korean adolescents was low, with rates of 23.4% for boys and 8.8% for girls [38], significantly lower than international standards [39]. Therefore, proactive efforts to promote physical activity are crucial for increasing handgrip strength among Korean children and adolescents.

This study highlights the significance of childhood handgrip strength as a preventive factor against MetS in adolescence from a public health standpoint. Handgrip strength is a key indicator of overall muscle strength, underscoring the need for regular physical activity and strength training beginning at an early age [40]. The World Health Organization and the Korea Ministry of Health and Welfare recommend that children aged 6 and older participate in physical activities, including strength training, at least 3 times a week. Activities like stair climbing, push-ups, and squats are beneficial for muscle strengthening [41,42].

This study has several limitations. First, the participants were selected from a specific hospital, which may limit the generalizability of the results. Second, the study utilized a cohort design, which introduces the potential for bias due to attrition in follow-up observations. However, no significant differences were observed in mean handgrip strength and anthropometry at ages 7-9 years between participants who continued in the follow-up at ages 13-15 years and those who did not. Third, the absence of handgrip strength measurements at ages 13-15 years constrains our ability to elucidate the impact of adolescent handgrip strength levels on the relationship between handgrip strength and metabolic outcomes. Lastly, the frequency of moderate physical activity, considered a confounding variable, was assessed using a questionnaire without an objective evaluation of actual physical fitness or cardiorespiratory fitness. Even with adjustments for changes in BMI to account for physical growth, residual confounding factors may still be present. Despite these limitations, the study’s strength lies in its evaluation of the association between childhood handgrip strength levels and MetS and insulin resistance in adolescence through a prospective cohort design.

Our findings revealed a significant inverse relationship between childhood handgrip strength and both MetS scores and insulin resistance during adolescence. These results offer valuable epidemiological evidence from a prospective study. Since MetS in adolescence can persist into adulthood, early prevention is essential. Additionally, handgrip strength, which acts as a surrogate marker for muscle strength, seems to be a practical indicator for assessing the risk of MetS and insulin resistance. This highlights the critical need for preventive strategies aimed at improving muscle strength in children.

## Figures and Tables

**Figure f5-epih-47-e2025001:**
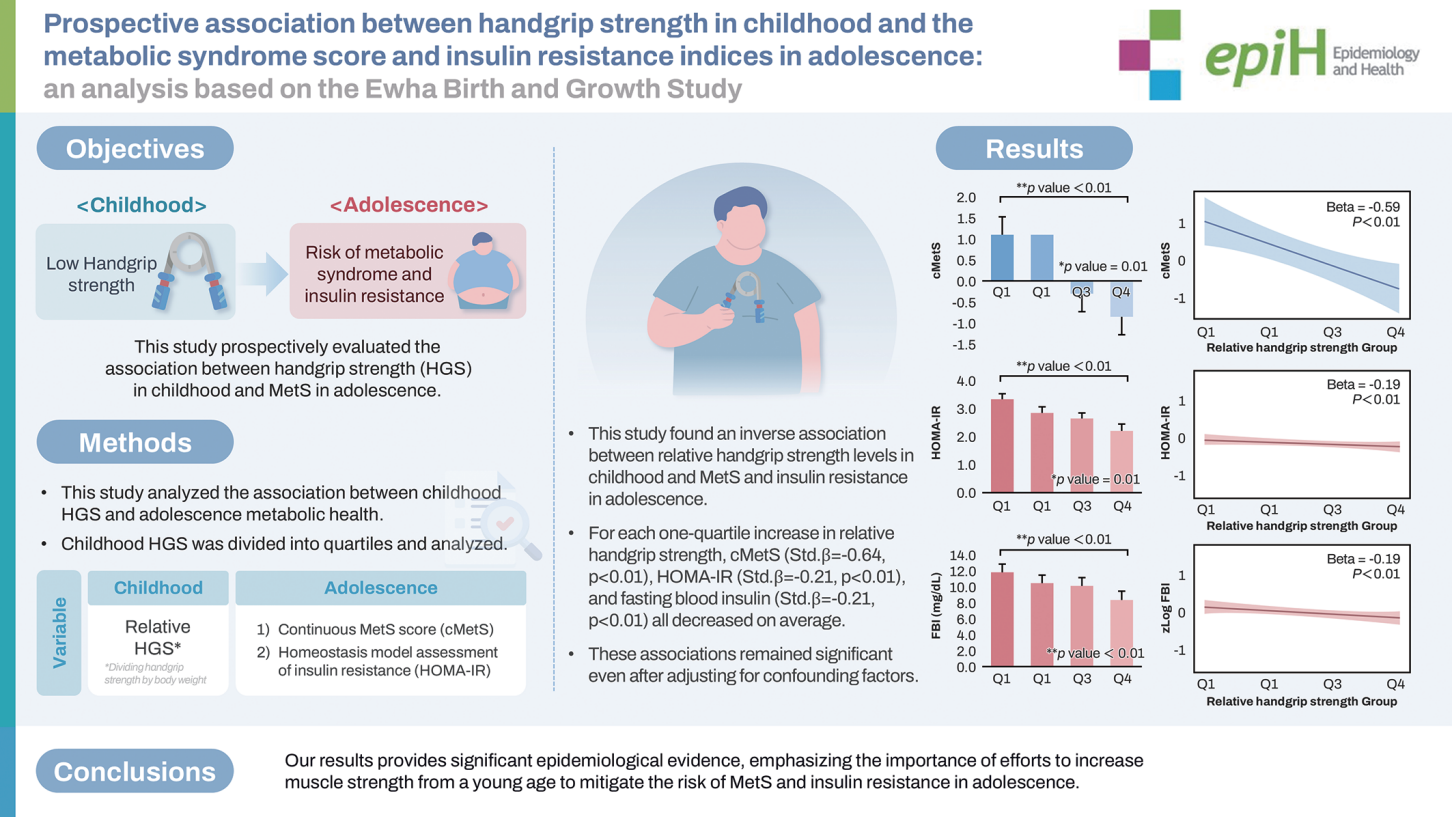


**Table 1.** Characteristics of the study participants

Characteristics	Total (n=219)	Boys (n=107, 48.9%)	Girls (n=112, 51.1%)	p-value
At 7-9 yr				
Age (yr)	7.42±0.69	7.39±0.70	7.44±0.69	0.63
Weight (kg)	26.42±6.02	26.79±6.35	26.06±5.69	0.37
Weight SDS	-0.35±1.11	-0.24±1.12	-0.42±1.10	0.22
cMetS	-0.00±3.07	0.03±3.02	-0.03±3.13	0.88
BMI (kg/m^2^)	16.44±2.33	16.52±2.47	16.35±2.20	0.59
BMI SDS	-0.36±1.13	-0.34±1.18	-0.39±1.08	0.65
MAP (mmHg)	73.57±7.06	74.37±7.26	72.79±6.82	0.10
SBP (mmHg)	101.77±10.32	103.08±10.35	100.53±10.19	0.07
DBP (mmHg)	59.46±6.65	60.02±7.03	58.93±6.25	0.23
FBG (mg/dL)	79.73±8.58	81.08±9.83	78.47±7.03	0.03
TG (mg/dL)	51.0 (36.0-80.0)	43.0 (32.0-69.0)	56.5 (39.0-93.5)	<0.01
HDL-C (mg/dL)	61.03±11.80	64.25±11.31	58.01±11.50	<0.01
HOMA-IR	1.43±1.04	1.24±0.57	1.61±1.31	<0.01
FBI (μU/mL)	6.7 (5.0-8.5)	6.2 (4.5-7.7)	7.5 (5.5-9.1)	<0.01
At 13-15 yr				
Age (yr)	13.25±0.54	13.21±0.50	13.28±0.59	0.40
Weight (kg)	53.67±10.71	55.78±12.23	51.66±8.61	<0.01
Weight SDS	-0.11±1.03	0.08±1.11	-0.31±0.90	<0.01
cMetS	0.10±3.06	0.08±3.12	0.12±3.02	0.92
BMI (kg/m^2^)	20.75±3.41	20.92±3.84	20.59±2.96	0.47
BMI SDS	-0.03±1.13	0.01±1.24	-0.06±1.01	0.56
MAP (mmHg)	81.95±8.59	83.03±8.82	80.92±8.27	0.07
SBP (mmHg)	110.56±11.48	112.36±11.61	108.84±11.13	0.02
DBP (mmHg)	67.65±8.39	68.36±8.84	66.96±7.91	0.22
FBG (mg/dL)	93.51±6.42	93.76±5.73	93.28±7.03	0.58
TG (mg/dL)	77.2 (51.0-88.0)	73.8 (45.0-85.0)	80.5 (54.5-90.5)	0.04
HDL-C (mg/dL)	51.54±9.46	51.39±8.88	51.68±10.02	0.82
HOMA-IR	2.67±1.58	2.56±1.61	2.78±1.54	0.30
FBI (μU/mL)	11.4 (7.5-13.9)	11.0 (6.4-12.9)	11.9 (7.9-14.3)	0.03

Values are presented as mean±standard deviation or median (interquartile range). SDS, standard deviation score; cMetS, continuous metabolic syndrome score; BMI, body mass index; MAP, mean arterial pressure; SBP, systolic blood pressure; DBP, diastolic blood pressure; FBG, fasting blood glucose; TG, triglyceride; HDL-C, high-density lipoprotein cholesterol; HOMA-IR, homeostasis model assessment of insulin resistance; FBI, fasting blood insulin.

**Table 2.** Univariate analysis of MetS components and insulin resistance indices in adolescence according to relative handgrip strength quartiles in childhood

Variables	Relative handgrip strength quartiles^1^				p-value
	Q1	Q2	Q3	Q4	
Total (n)	55	55	55	54	
MetS score and its components					
cMetS	1.15±3.32	0.29±2.93	-0.27±3.25	-0.80±2.41	<0.01
BMI (kg/m^2^)	22.65±3.52	21.45±3.60	19.96±2.85	18.91±2.34	<0.01
MAP (mmHg)	83.01±8.74	81.93±9.10	80.61±9.56	82.26±6.70	0.53
SBP (mmHg)	111.21±11.72	110.65±11.52	109.73±13.69	110.64±8.63	0.93
DBP (mmHg)	68.91±8.74	67.56±9.11	66.05±8.74	68.06±6.69	0.34
FBG (mg/dL)	94.07±8.93	93.31±4.79	93.49±5.78	93.17±5.51	0.89
Log TG^2^	74.0 (54.0-91.0)	72.0 (51.0-94.0)	68.0 (51.0-92.0)	61.5 (48.0-77.0)	0.32
HDL-C (mg/dL)	49.95±10.59	51.16±8.27	52.18±9.48	52.89±9.34	0.39
Insulin resistance index					
HOMA-IR	3.24±2.15	2.69±1.48	2.58±1.27	2.17±1.01	<0.01
Log FBI^2^	12.0 (7.9-16.3)	9.6 (7.5-14.5)	9.9 (7.3-13.5)	8.7 (6.1-11.9)	<0.01
Boys (n)	27	27	27	26	
MetS score and its components					
cMetS	0.87±3.25	0.82±3.30	-0.77±3.02	-0.65±2.65	0.08
BMI (kg/m^2^)	22.93±4.30	22.08±4.03	19.70±2.89	18.91±2.48	<0.01
MAP (mmHg)	84.45±9.38	83.70±8.84	80.96±9.53	82.99±7.41	0.51
SBP (mmHg)	113.83±12.67	112.59±10.95	111.11±12.76	111.87±10.30	0.85
DBP (mmHg)	69.76±9.60	69.26±8.87	65.89±9.56	68.56±7.01	0.38
FBG (mg/dL)	95.78±6.49	93.37±4.99	92.56±6.01	93.31±5.08	0.19
LogTG^2^	59.0 (44.0-79.0)	73.0 (46.0-102.0)	60.0 (47.0-87.0)	59.5 (42.0-77.0)	0.60
HDL-C (mg/dL)	51.96±11.38	49.04±7.02	52.52±8.41	52.08±8.17	0.46
Insulin resistance index					
HOMA-IR	3.08±1.87	2.69±1.71	2.44±1.53	2.00±1.10	0.10
Log FBI^2^	12.0 (7.9-16.3)	9.6 (7.5-14.5)	9.9 (7.3-13.5)	8.7 (6.1-11.9)	0.04
Girls (n)	28	28	28	28	
MetS score and its components					
cMetS	1.42±3.41	-0.21±2.47	0.21±3.44	-0.95±2.20	0.03
BMI (kg/m^2^)	22.38±2.63	20.85±3.08	20.22±2.84	18.90±2.25	<0.01
MAP (mmHg)	81.62±7.99	80.21±9.18	80.27±9.74	81.57±6.01	0.87
SBP (mmHg)	108.68±10.33	108.79±11.94	108.39±14.64	109.50±6.72	0.99
DBP (mmHg)	68.09±7.90	65.93±9.20	66.21±8.06	67.61±6.47	0.69
FBG (mg/dL)	92.43±10.65	93.25±4.69	94.39±5.51	93.04±5.98	0.77
LogTG^2^	87.0 (61.5-109.0)	68.9 (52.5-90.5)	69.0 (56.0-93.0)	62.5 (53.5-74.0)	0.04
HDL-C (mg/dL)	48.00±9.57	53.21±8.97	51.86±10.55	53.64±10.41	0.14
Insulin resistance indices					
HOMA-IR	3.39±2.41	2.68±1.26	2.71±0.98	2.33±0.92	0.07
Log FBI^2^	14.0 (8.3-17.4)	10.7 (7.7-15.5)	10.8 (8.7-13.4)	9.6 (7.5-12.4)	0.12

Values are presented as mean±standard deviation or median (interquartile range). MetS, metabolic syndrome; cMetS, continuous metabolic syndrome score; BMI, body mass index; MAP, mean arterial pressure; SBP, systolic blood pressure; DBP, diastolic blood pressure; FBG, fasting blood glucose; TG, triglyceride; HDL-C, high-density lipoprotein cholesterol; HOMA-IR, homeostasis model assessment of insulin resistance; FBI, fasting blood insulin.

1 Relative handgrip strength quartiles were defined taking sex into account; The range of each quartile is as follows; first quartile (Q1<0.35), second quartile (0.35≤Q2<0.40), third quartile (0.40≤Q3<0.48), and fourth quartile (Q4≥0.48) in boys and first quartile (Q1<0.33), second quartile (0.33≤Q2<0.38), third quartile (0.38≤Q3<0.43), and fourth quartile (Q4≥0.43) in girls.

2 Statistical analysis was performed using the Kruskal-Wallis test.

## Supplementary materials

Supplementary Material 1. Handgrip strength and anthropometric differences in 7 to 9 year old follow-up participants based on study inclusion.

Supplementary Material 2. Association of relative handgrip strength quartiles a in childhood with metabolic syndrome and insulin resistance in adolescence.

Supplementary Material 3. Association of HGS-to-BFM ratio quartiles a in childhood with metabolic syndrome and insulin resistance in adolescence.
