# Supplementary material for: Prospective association between handgrip strength in childhood and the metabolic syndrome score and insulin resistance indices in adolescence: an analysis based on the Ewha Birth and Growth Study
Source: Epidemiol Health. 2025 Jan 2;47:e2025001. doi: 10.4178/epih.e2025001 (PMC11920678; doi:10.4178/epih.e2025001)
Supplement: Supplementary Material 1. — Handgrip strength and anthropometric differences in 7 to 9 year old follow-up participants based on study inclusion. [file epih-47-e2025001-Supplementary-1.docx]

**Supplementary Material**

Supplementary Material 1. Handgrip strength and anthropometric differences in 7 to 9 year old follow-up participants based on study inclusion.

| Variables | Included in this study  (n = 219) | Excluded in this study  (n = 365) | *P* value |
| --- | --- | --- | --- |
| Height (cm) | 126.10 ± 6.85 | 127.00 ± 6.81 | 0.14 |
| Height SDS | -0.15 ± 1.02 | -0.20 ± 1.08 | 0.60 |
| Weight (kg) | 26.42 ± 6.02 | 26.93 ± 6.07 | 0.32 |
| Weight SDS | -0.35 ± 1.11 | -0.38 ± 1.22 | 0.65 |
| BMI (kg/m^2^) | 16.44 ± 2.33 | 16.52 ± 2.51 | 0.69 |
| BMI SDS | -0.36 ± 1.13 | -0.39 ± 1.24 | 0.75 |
| Handgrip strength (kg) | 10.47 ± 3.45 | 10.81 ± 3.91 | 0.27 |
| Relative handgrip strength ^a^ | 0.40 ± 0.12 | 0.41 ± 0.14 | 0.78 |

SDS, Standard Deviation Score; BMI, body mass index.

^a^ Relative handgrip strength was calculated by dividing handgrip strength (kg) by body weight (kg).
