# Supplementary material for: Prospective association between handgrip strength in childhood and the metabolic syndrome score and insulin resistance indices in adolescence: an analysis based on the Ewha Birth and Growth Study
Source: Epidemiol Health. 2025 Jan 2;47:e2025001. doi: 10.4178/epih.e2025001 (PMC11920678; doi:10.4178/epih.e2025001)
Supplement: Supplementary Material 2. — Association of relative handgrip strength quartiles a in childhood with metabolic syndrome and insulin resistance in adolescence. [file epih-47-e2025001-Supplementary-2.docx]

Supplementary Material 2. Association of relative handgrip strength quartiles ^a^ in childhood with metabolic syndrome and insulin resistance in adolescence.

| Variables | Crude model | | | | | | Adjusted model ^b^ | | | | | |
| --- | --- | --- | --- | --- | --- | --- | --- | --- | --- | --- | --- | --- |
|  | Total | | Boys | | Girls | | Total | | Boys | | Girls | |
|  | *β* (95% CI) | *P* value | *β* (95% CI) | *P* value | *β* (95% CI) | *P* value | *β* (95% CI) | *P* value | *β* (95% CI) | *P* value | *β* (95% CI) | *P* value |
| Metabolic syndrome score and its components | | | | | | | | | | | | |
| cMetS | -0.64  (-1.00, -0.29) | < 0.01 | -0.62  (-1.14, -0.09) | 0.02 | -0.67  (-1.16, -0.18) | <0.01 | -0.59  (-0.93, -0.25) | < 0.01 | -0.48  (-0.96, 0.00) | 0.05 | -0.69  (-1.19, -0.19) | < 0.01 |
| zBMI | -0.37  (-0.48, -0.27) | < 0.01 | -0.38  (-0.53, -0.22) | <0.01 | -0.37  (-0.53, -0.22) | <0.01 | -0.35  (-0.45, -0.26) | < 0.01 | -0.34  (-0.47, -0.21) | < 0.01 | -0.36  (-0.49, -0.23) | < 0.01 |
| zMAP | -0.04  (-0.16, 0.08) | 0.50 | -0.08  (-0.25, 0.09) | 0.35 | 0.00  (-0.17, 0.17) | 0.99 | -0.03  (-0.15, 0.09) | 0.60 | -0.06  (-0.24, 0.13) | 0.53 | -0.02  (-0.19, 0.15) | 0.83 |
| zSBP | -0.02  (-0.14, 0.10) | 0.72 | -0.06  (-0.24, 0.11) | 0.46 | 0.02  (-0.15, 0.19) | 0.83 | 0.00  (-0.12, 0.12) | 0.95 | -0.05  (-0.22, 0.13) | 0.61 | 0.02  (-0.15, 0.18) | 0.84 |
| zDBP | -0.05  (-0.17, 0.07) | 0.44 | -0.08  (-0.25, 0.09) | 0.36 | -0.01  (-0.18, 0.15) | 0.86 | -0.05  (-0.17, 0.08) | 0.45 | -0.06  (-0.24, 0.13) | 0.56 | -0.04  (-0.21, 0.13) | 0.63 |
| zFBG | -0.05  (-0.17, 0.07) | 0.42 | -0.15  (-0.32, 0.03) | 0.10 | 0.04  (-0.13, 0.21) | 0.62 | -0.07  (-0.19, 0.06) | 0.29 | -0.13  (-0.31, 0.06) | 0.17 | 0.00  (-0.18, 0.17) | 0.97 |
| zlogTG ^c^ | -0.09  (-0.21, 0.03) | 0.12 | 0.01  (-0.17, 0.18) | 0.95 | -0.19  (-0.35, -0.02) | 0.03 | -0.06  (-0.18, 0.06) | 0.34 | 0.07  (-0.10, 0.24) | 0.43 | -0.17  (-0.34, 0.01) | 0.06 |
| zHDL-C | 0.10  (-0.02, 0.22) | 0.10 | 0.04  (-0.13, 0.22) | 0.62 | 0.16  (-0.01, 0.32) | 0.07 | 0.10  (-0.03, 0.22) | 0.13 | 0.04  (-0.14, 0.22) | 0.67 | 0.15  (-0.02, 0.32) | 0.09 |
| Insulin resistance index | | | | | | | | | | | | |
| zHOMA-IR | -0.21  (-0.33, -0.09) | < 0.01 | -0.22  (-0.38, -0.05) | 0.01 | -0.21  (-0.37, -0.04) | 0.01 | -0.19  (-0.31, -0.08) | < 0.01 | -0.17  (-0.32, -0.02) | 0.03 | -0.21  (-0.38, -0.03) | 0.02 |
| zlogFBI ^c^ | -0.21  (-0.32, -0.09) | < 0.01 | -0.24  (-0.41, -0.07) | < 0.01 | -0.18  (-0.34, -0.01) | 0.03 | -0.19  (-0.30, -0.07) | < 0.01 | -0.20  (-0.35, -0.06) | 0.01 | -0.16  (-0.34, 0.01) | 0.06 |

95% CI, 95% confidence intervals; cMetS, continuous metabolic syndrome score; BMI, body mass index; MAP, mean arterial pressure; SBP, systolic blood pressure; DBP, diastolic blood pressure; FBG, fasting blood glucose; TG, triglyceride; HDL-C, high-density lipoprotein-cholesterol; HOMA-IR, homeostasis model assessment of insulin resistance; FBI, fasting blood insulin.

Beta coefficients and 95% CI were obtained from the linear regression model by assigning the median to each quartile and treating it as a continuous variable.

^a^ Relative handgrip strength quartiles were defined taking sex into account. The range of each quartile is as follows; 1^st^ quartile (Q1 < 0.35), 2^nd^ quartile (0.35 ≤ Q2 < 0.40), 3^rd^ quartile (0.40 ≤ Q3 < 0.48), and 4^th^ quartile (Q4 ≥ 0.48) in boys and 1^st^ quartile (Q1 < 0.33), 2^nd^ quartile (0.33 ≤ Q2 < 0.38), 3^rd^ quartile (0.38 ≤ Q3 < 0.43), and 4^th^ quartile (Q4 ≥ 0.43) in girls.

^b^ Adjusted for sex, age, monthly household income, the mother’s education level, moderate physical activity at the age of 13-15 years, and change in BMI from the age of 7-9 to 13-15.

^c^ Log transformation applied due to non-normal distributed.
