# Supplementary material for: Prospective association between handgrip strength in childhood and the metabolic syndrome score and insulin resistance indices in adolescence: an analysis based on the Ewha Birth and Growth Study
Source: Epidemiol Health. 2025 Jan 2;47:e2025001. doi: 10.4178/epih.e2025001 (PMC11920678; doi:10.4178/epih.e2025001)
Supplement: Supplementary Material 3. — Association of HGS-to-BFM ratio quartiles a in childhood with metabolic syndrome and insulin resistance in adolescence. [file epih-47-e2025001-Supplementary-3.docx]

Supplementary Material 3. Association of HGS-to-BFM ratio quartiles ^a^ in childhood with metabolic syndrome and insulin resistance in adolescence.

| Variables | Crude model | | Adjusted model ^b^ | |
| --- | --- | --- | --- | --- |
|  | *β* (95% CI) | *P* value | *β* (95% CI) | *P* value |
| Metabolic syndrome score and its components | | | | |
| cMetS | -0.73 (-1.11, -0.35) | < 0.01 | -0.83 (-1.17, -0.49) | < 0.01 |
| BMI (kg/m^2^) | -1.65 (-2.05, -1.24) | < 0.01 | -1.62 (-1.94, -1.29) | < 0.01 |
| MAP (mmHg) | -0.02 (-1.23, 1.18) | 0.97 | -0.29 (-1.47, 0.89) | 0.63 |
| SBP (mmHg) | -0.08 (-1.71, 1.56) | 0.93 | -0.53 (-2.07, 1.02) | 0.50 |
| DBP (mmHg) | 0.00 (-1.20, 1.20) | 1.00 | -0.17 (-1.39, 1.05) | 0.78 |
| FBG (mg/dL) | 0.28 (-0.55, 1.11) | 0.51 | 0.00 (-0.86, 0.86) | 1.00 |
| Log TG ^c^ | -0.05 (-0.11, 0.00) | 0.07 | -0.06 (-0.12, 0.00) | 0.07 |
| HDL-C (mg/dL) | 1.73 (0.38, 3.08) | 0.01 | 1.96 (0.58, 3.34) | < 0.01 |
| Insulin resistance index | | | | |
| HOMA-IR | -0.33 (-0.53, -0.13) | < 0.01 | -0.36 (-0.56, -0.15) | < 0.01 |
| Log FBI ^c^ | -0.09 (-0.16, -0.03) | < 0.01 | -0.10 (-0.17, -0.03) | < 0.01 |

HGS, handgrip strength; BFM, body fat mass; 95% CI, 95% confidence intervals; cMetS, continuous metabolic syndrome score; BMI, body mass index; MAP, mean arterial pressure; SBP, systolic blood pressure; DBP, diastolic blood pressure; FBG, fasting blood glucose; TG, triglyceride; HDL-C, high-density lipoprotein-cholesterol; HOMA-IR, homeostasis model assessment of insulin resistance; FBI, fasting blood insulin.

Beta coefficients and 95% CI were obtained from the linear regression model by assigning the median to each quartile and treating it as a continuous variable.

^a^ HGS-to-BFM ratio quartiles were defined taking sex into account. The range of each quartile is as follows; 1^st^ quartile (Q1 < 1.48), 2^nd^ quartile (1.48 ≤ Q2 < 2.21), 3^rd^ quartile (2.21 ≤ Q3 < 2.71), and 4^th^ quartile (Q4 ≥ 2.71) in boys and 1^st^ quartile (Q1 < 1.14), 2^nd^ quartile (1.14 ≤ Q2 < 1.57), 3^rd^ quartile (1.57 ≤ Q3 < 2.15), and 4^th^ quartile (Q4 ≥ 2.15) in girls.

^b^ Adjusted for sex, age, monthly household income, the mother’s education level, moderate physical activity at the age of 13-15 years, and change in BMI from ages 7-9 to 13-15.

^c^ Log transformation applied due to non-normal distributed.
